# Cystic-solid hemangioblastoma at the cerebellopontine angle: A case report

**DOI:** 10.1097/MD.0000000000018871

**Published:** 2020-01-17

**Authors:** Zhigang Lan, Seidu A. Richard, Yuekang Zhang

**Affiliations:** a Department of Neurosurgery, West China Hospital, Sichuan University, Chengdu, Sichuan, P. R. China; b Department of Medicine, Princefield University, Ghana, West Africa.

**Keywords:** cerebellopontine angle, cystic-solid, hemangioblastoma, hypervascular, retro-sigmoid

## Abstract

**Rationale:**

Hemangioblastomas (HMGs) originating from the cerebellopontine angle (CPA) are extremely uncommon. Nevertheless, the cystic-solid form of this lesion at the above location is even rarer.

**Patient concerns:**

We present a 31-years old male with a right ear hearing loss of 3 months duration. He did not experience earache or discharge before the hearing loss. He; however, experienced visual acuity and dizziness. General physical examination did not yield much.

**Diagnoses:**

Computed tomography and magnetic resonance imaging revealed a cystic-solid mass at right CPA. We initial misdiagnosed the lesion as an acoustic neuroma with cystic changes. Immunohistochemistry studies revealed HMG.

**Interventions:**

The lesion was total surgical resection via the retro-sigmoid approach.

**Outcomes:**

The patient's symptomatology resolved after the surgery. Two years follow-up show no recurrence of the lesion and the patient is well.

**Lesions:**

Identification of feeding arteries and electro-coagulating them during the operation minimized intraoperative bleeding. The tumor should usually be dissected out whole and not piece meal fashion. Pre-operative CTA is very useful in outlining the vasculature of the tumor.

## 1 Introduction

Hemangioblastomas (HMBs) originating from the cerebellopontine angle (CPA) are extremely uncommon.^[1–3]^ HMBs may be seen on radiological images as solid, solid-cystic, or predominantly cystic with a small mural.^[4,5]^ The cystic-solid form of this lesion at the above location is even rarer. The cystic forms of HMBs are often made up of a tiny vascularized nidus and a very large cystic portion.^[3,4]^ The solid forms are often uniformly solid while the cystic-solid forms compose of a large cystic portion as well as a large solid portion.^[4,6]^ HMBs are usually hypervascular and the solid cerebellar types often originates adjacent the brain stem.^[7,8]^

Computed tomography (CT) and magnetic resonance imaging (MRI) are often the gold-standard radiological imaging modalities used in identifying HMBs although they are often mistaken for other CPA neoplasms.^[2,4,5,9]^ They often appear as cystic lesions with a nodule of extreme contrast enhancement or a solid lesion.^[6,9]^ Open surgery is the gold-standard treatment modality for HMBs.^[10]^ Radiosurgery has also proven to be a secure alternative treatment option. The solid forms of HMBs at the CPA often poses major surgical challenge, because very large ones often present with profuse bleeding intraoperatively.^[6]^ The complex neurovascular structures at the CPA often make navigation more difficult. Our case is a cystic-solid variety of HMB at the CPA which we operated on with no major complications as well as neurological deficits.

## 2 Case report

We present a 31-years old male with a right ear hearing loss of 3 months duration. He did not experience earache or discharge before the hearing loss. He; however, experienced visual acuity and dizziness. General physical examination did not yield much. Cranial nerve examinations were unremarkable. We did not observe any cerebellar signs and symptoms. We did not observe any discharges from the ear and otoscopic examination did not reveal same. Rinne and Weber tests demonstrated sensorineural hearing loss. Fundoscopic examination did not yield much. Routine laboratory investigations were within normal rangers.

**Figure 1:**
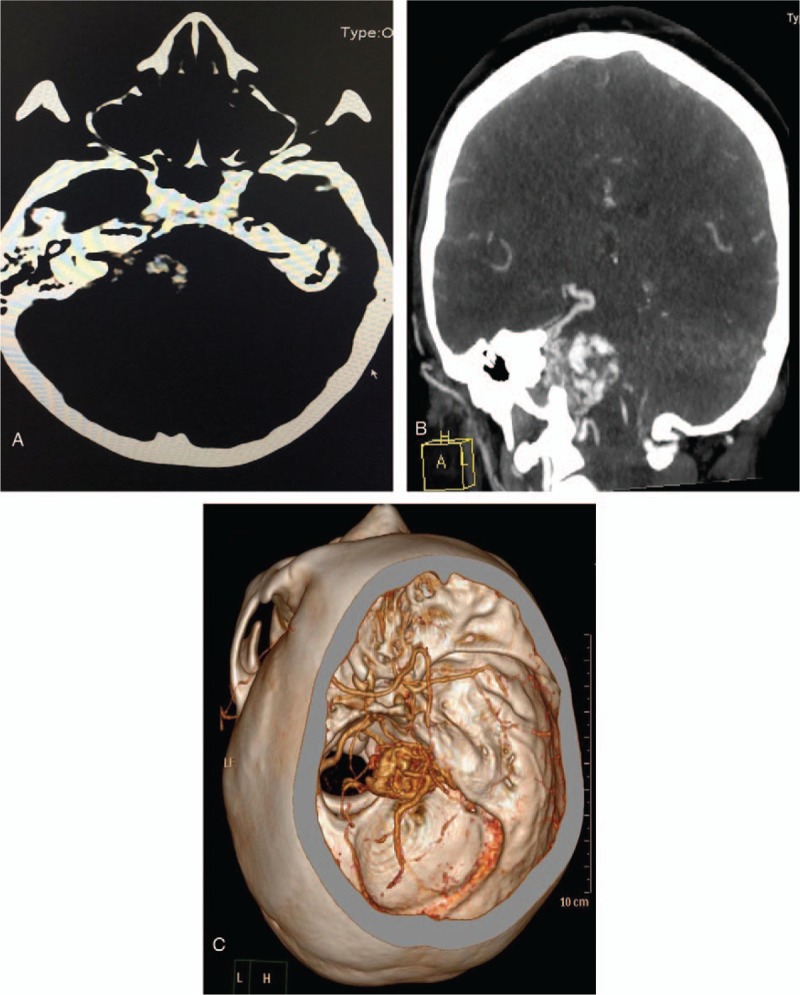
(A) A preoperative CT image showing expansion of the right auditory canal. (B and C) Preoperative CTA images showing the vasculature of the tumor. CT = computed tomography, CTA = computed tomography angiography.

**Figure 2:**
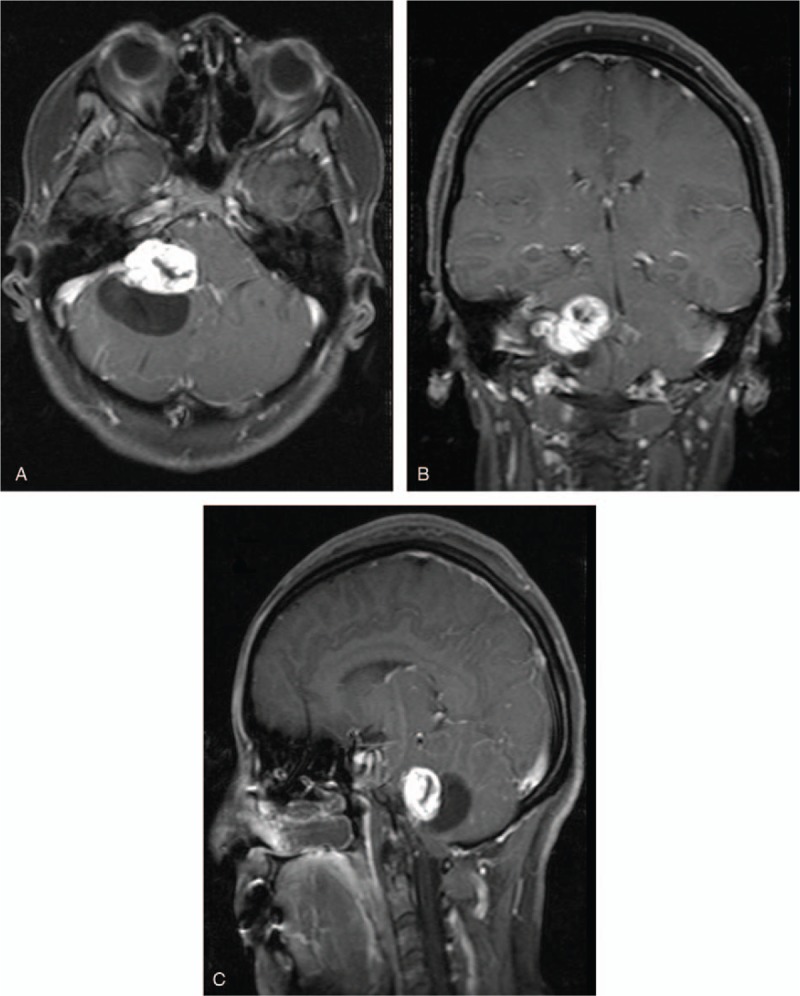
(A–C) MRIs showing a cystic-solid tumor. MRI = magnetic resonance imaging.

CT revealed a cystic-solid mass at right CPA measuring about 5.3 × 4.3 cm with slightly dense regions. We noticed right auditory canal expansion (Fig. 1A). Enhanced CT demonstrated partial heterogeneous enhancement. We noticed thick and enlarged blood vessels all-round the lesion most of which drained into an enlarged great cerebral vein. Computed tomography angiography (CTA) also showed (Fig. 1B and C) a right CPA mass encasing the right vertebral artery (VA). The distal lumen of right VA was narrow due to compressive effect of the lesion. The right anterior cerebral artery was also narrow. The bilateral internal carotid arteries were normal. MRI also revealed a cystic-solid right CPA lesion (Fig. 2A–C) measuring about 5 × 4.0 × 3.5 cm. The solid part of the lesion demonstrated mixed signals intensities on T1 and T2-weighted images. We observed heterogeneous enhanced images on enhancement MRI. The auditory canal was slightly enlarged. The mass was compressing the cerebellum as well as the fourth ventricle. We did not observe hydrocephalus. Our working diagnosis was an acoustic neuroma with cystic changes.

We totally resected the lesion via the retro-sigmoid approach. Intraoperatively, we saw a cystic-solid tumor which was red in color and composed of vascular tissue. We saw a multitude of thick feeding arteries and veins all around the solid portion. The solid part was tough in consistency with clear boundaries. It was bleeding profusely on touch. The solid portion was adhered to the trigeminal nerve, acoustic nerve, facial nerve as well as the brain stem. The tumor originated from the cerebellar pial vessels and took an intra-axial route. We first and foremost identified the feeding arteries and electro-coagulated them to minimize intraoperative bleeding therefore blood loss was minimal (<200 mls). We then carefully resected of the tumor by separating it from the above vital structures. We utilized electromyographic (EMG) and auditory brainstem responses (ABRs) to monitor the cranial nerves above. All cranial nerves functions were intact after the operation.

**Figure 3:**
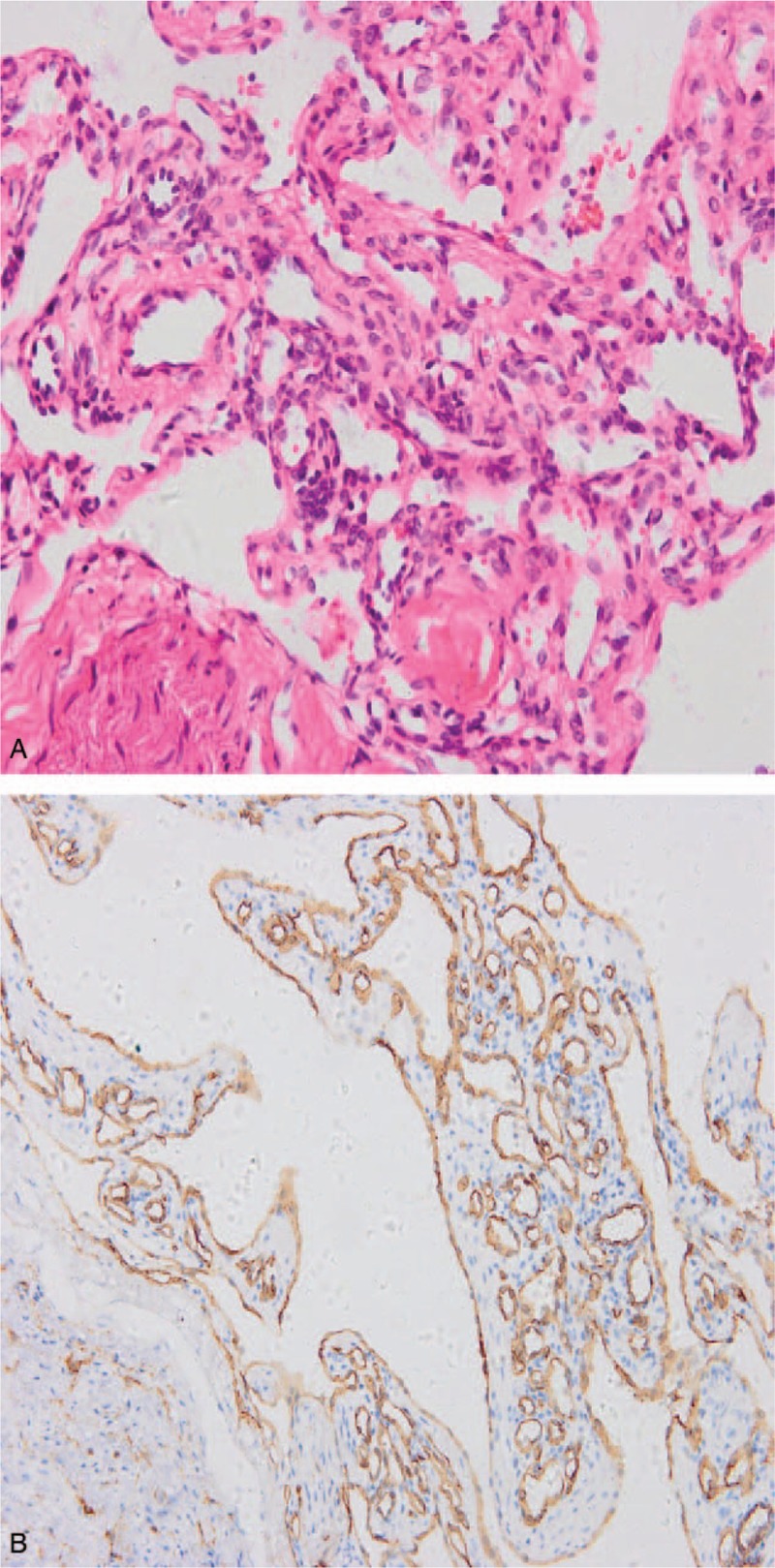
(A and B) Histochemical staining of the tumor after surgical resection.

**Figure 4:**
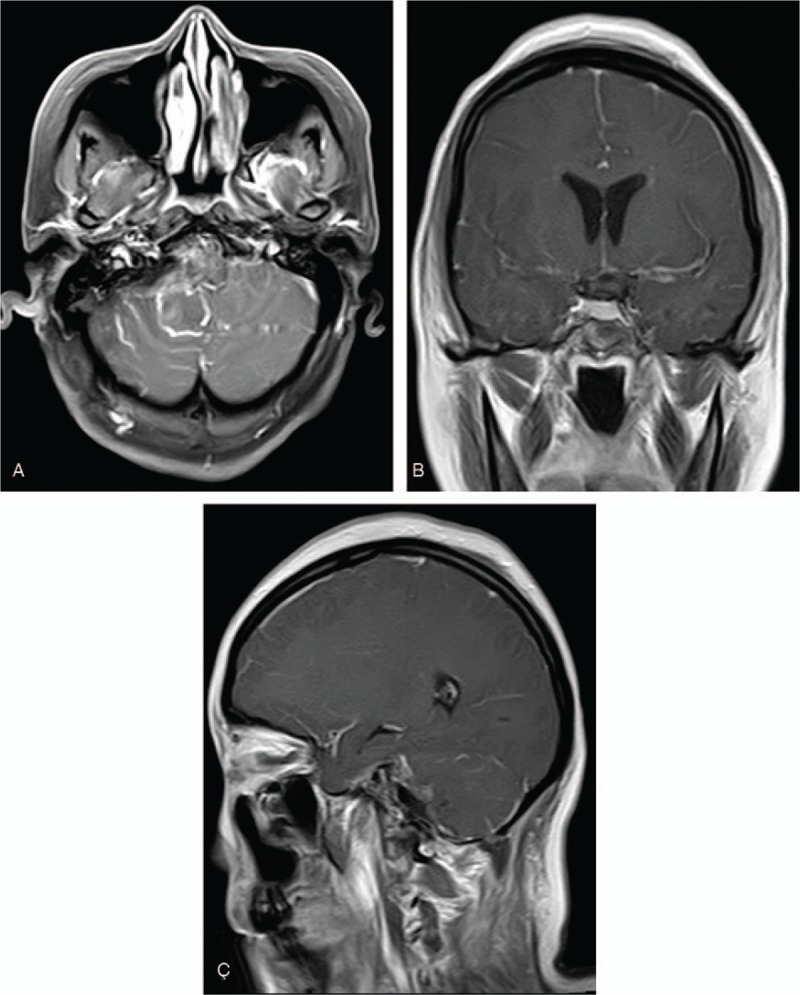
(A–C) Postoperative MRIs showing total resection of the tumor. MRIs = magnetic resonance imaging.

On histochemical staining, we observed intracytoplasmic glycogen within the foamy cytoplasm (Fig. 3A and B). Immunohistochemistry studies revealed that, the tumor cells were D2–40 (+), carbonic anhydrase (CAIX) (+ ), neuron-specific enolase (NSE) (±), Inhibin- α (+),brahma related gene-1 (BRG1) (+), Ki67 (+, 20%–30%), glial fibrillary acidic proteins (GFAP) (−), S-100 (−), CK (−), epithelial membrane antigen (−), P63 (−), CD34 (−), signal transducer and activator of transcription 6 (−), desmin (−), smooth muscle actin (−), thyroid transcription factor 1 (−), chromogranin A (−), synaptophysin (−). These finding are consistent with the diagnosis of HMB. Postoperative MRI demonstrated total resection of the tumor (Fig. 4A–C). Two years follow-up revealed no recurrence of the lesion and the patient is well. His right sided hearing loss was restored after the operation.

## 3 Discussion

HGMs constitutes of about 1.5% to 2.5% of all neoplasms arising intracranially and 7% to 12% of all posterior fossa neoplasms.^[1,10,11]^ These lesions are often seen in patients in their 30's or 40's.^[10]^ Nevertheless, the sexual predisposition of CPA HGMs has not been reported. HGMs often exist primarily as cystic with a tiny mural, solid, or cystic-solid.^[4]^ Our case was the cystic-solid type which mislead us in the diagnosis. HGMs usually have a vascularized nidus which is made up of stromal cells encased by abundant capillaries.^[4,5]^

Byung et al are of the view that, their lesions in their patient originate from the cerebellar pial vessels and took an intra-axial route^[12]^ as we also observed in our case. Nevertheless, the neoplasm developed at the CPA, an extra-axial site, instead of the cerebellar parenchyme.^[12]^ HMBs arises mostly in the cerebellum, brainstem, as well as the spinal cord.^[4,13]^ At the CPA, Hemangioblastoma (HMGs) are often misdiagnosed due to the peculiarity of the site, clinical presentation as well as imaging characteristics.^[4]^ The unusual cystic-solid nature of the lesion couples with the symptomatology initially make us misdiagnose the lesion as an acoustic neuroma with cystic changes.

Hearing loss, tinnitus, ataxia, as well as vertigo are the usual symptomatology in patients with HMGs.^[4]^ In most cases, this tumor is often misdiagnosed because these signs and symptom are typical presentation of most CPA tumors.^[4]^ CT and MRI are very useful in the definitive diagnoses of this tumor.^[9]^ On both CT and MRI, HMGs are frequently seen as a cystic mass with a small hypervascular mural nodule.^[9]^ The cysts typically have smooth boundaries as well as homogeneous cerebrospinal fluid (CSF)-like signal intensity.^[9]^

The solid variant is usually seen as a solid mass without any cystic constituent.^[14,15]^ Our case is the cystic-solid variant which was seen on both CT and MRI as a large cyst portion and a large solid portion. In the cystic type, the nodules are often seen as hypointense images on T1-weighted and hyperintense images on T2-weighted on MRI.^[14]^ The lesion enhances intensely on contrast.^[14]^ In most cases, high-flow vessels can be seen as flow voids at the periphery of the mass due to the hypervascularity of the tumor. Edema is mostly insignificant or nonexistent around lesion in all the tumor variants.^[14]^

Surgery via the middle fossa, retro-sigmoid or transtemporal approach is still the most preferred treatment modality for CPA HMGs.^[6,10]^ It is advocated that as much as possible, solid HMGs should be dissected and excised whole instead of piecemeal manner because of associated life threating hemorrhage.^[6]^ Total resection is achievable with wide field surgical exposure as well as circumferential dissection and devascularization.^[6]^ Nevertheless, restricted access via the retro-sigmoid surgical exposure as well as the complex neurovascular anatomy of the area of the CPA usually make total excision problematic.^[6]^ We achieved total resection of the tumor via the retro-sigmoid approach. Radiosurgery is a secure alternative treatment for residual HMGs after surgical resection.^[7]^ This treatment modality is usually an adjuvant therapy for residual tumor after surgical resection.^[16]^ Radiosurgery is appropriate during tumor recurrence as well as unresectable or deep-rooted tumors at the CPA.^[7]^ We did not adapt the radiosurgery treatment because we achieved total resection and no recurrence of the lesion.

Kamitani et al established that preoperative radiosurgery as well as embolization of the feeding vessels are very advantageous in the treatment of solid HMGs with hypervascularity.^[7]^ They indicated that radiosurgery may diminish the possibility of extreme intraoperative bleeding during surgical resection.^[7]^ Nevertheless, there are hypothetical life threating complications accompanying preoperative embolization that cannot be overlooked.^[7]^ Corneliusc et al observed poor prognosis, acute tumor hemorrhage as well as death as cardinal complication associated with preoperative embolization for HMGs.^[17]^ Furthermore, tumor engorgement, as well as vessel occlusion resulting in acute infarction, has been associated with preoperative embolization for HMGs.^[18,19]^

Another very promising treatment modality for CPA HMGs is endovascular selective chemotherapy.^[9]^ Chemotherapy is possible because of the similarity of histogenesis of HMGs.^[9]^ Selective intra-arterial chemotherapy may be effective in the treatment of HMGs because of their ability to regulate the proliferation of endothelial cells, smooth muscle cells, as well as fibroblasts.^[9]^ Cheng et al observed facial paresis, lower cranial nerves deficits, abducens nerve paralysis, facial hypesthesia, cerebellar hemorrhage, CSF leakage, as well as pseudo-meningocele as the postoperative complication in patients who were surgical treated.^[4]^ We did not observe any such complication after surgical resection of the lesions in our patient. We utilized EMG and ABRs to monitor the cranial nerves. After our operation, we observe the cranial nerves functions and found them intact. Though HMGs are considered as histologically benign tumors, recurrence of the lesions have been reported after total resection.^[20,21]^ We did not observe any recurrence of the tumor 2 years after surgical resection. Nevertheless, recurrence rate of HMGs is about 10% to 27%.^[20]^

Histochemically, on periodic acid–Schiff staining, characteristic intracytoplasmic glycogen within the foamy cytoplasm are often observed.^[2,11]^ On the other hand, protein inhibin-α, a member of the transforming growth factor β family is usually strongly positive in most HMBs on immunostaining.^[2]^ Also, epidermal growth factor receptor and platelet-derived growth factor are strongly positive on immunostaining. Furthermore, focal areas of immunoreactivity with S-100 protein, NSE as well as GFAP have also been reported.^[2]^ We observer positivity for NSE, Inhibin- α, CAIX, podoplanin (D2–40), as well as BRG on immunostaining which confirmed the diagnosis of HMG.

## 4 Conclusion

Cystic-solid form of HMG at the CPA is very rare. The symptomology as well as radiological findings of HMGs at the CPA often makes these lesions misdiagnosed for other CPA tumors. CTA is very helpful in outlining the vasculature of the lesions during preoperative assessment. The tumor should be dissected out whole as much as possible to avoid life threating bleeding. Feeding arteries should be identifies and electro-coagulated during the operation to minimize intraoperative bleeding.

## Author contributions

**Conceptualization:** Zhigang Lan, Seidu A. Richard, Yuekang Zhang.

**Data curation:** Zhigang Lan, Seidu A. Richard, Yuekang Zhang.

**Funding acquisition:** Yuekang Zhang.

**Methodology:** Seidu A. Richard.

**Writing – original draft:** Seidu A. Richard.

**Writing – review and editing:** Zhigang Lan, Seidu A. Richard, Yuekang Zhang.
